# Recombinant portal protein from *Staphylococcus epidermidis* bacteriophage CNPH82 is a 13-subunit oligomer

**DOI:** 10.1107/S1744309112037645

**Published:** 2012-09-29

**Authors:** Weisha Luan, Jochen Fesseler, Maria Chechik, Carina R. Buttner, Alfred A. Antson, Callum Smits

**Affiliations:** a York Structural Biology Laboratory, Department of Chemistry, University of York, York, YO10 5DD, England

**Keywords:** portal protein, DNA translocation, bacteriophage CNPH82, oligomeric state

## Abstract

The portal protein cn3 of bacteriophage CNPH82 is predicted to serve as a gateway for translocation of viral genome into preformed pro-capsid, like portal proteins from other double-stranded DNA tailed bacteriophages. The host of bacteriophage CNPH82 is the opportunistic human pathogenic bacterium *Staphylococcus epidermidis*, a major cause of nosocomial infections. The portal protein of this phage has been cloned, overexpressed and purified. Size-exclusion chromatography–multi-angle laser light scattering analysis has indicated that the portal protein contains ∼13 subunits. Crystals of the portal protein, diffracting to 4.2 Å, have been obtained. These crystals belong to the space group *C*222_1_ with the unit-cell parameters of *a* = 252.4, *b* = 367.0, *c* = 175.5 Å. The self-rotation function revealed the presence of a single 13-subunit oligomer in the asymmetric unit.

## Abstract 2

Crystals of the portal protein from *Staphylococcus epidermidis* bacteriophage CNPH82, diffracting to ∼4.2 Å resolution, have been obtained. The protein is a 13-subunit oligomer both in solution and in the crystal.

## 1. Introduction

CNPH82 is a bacteriophage infecting the opportunistic pathogen *Staphylococcus epidermidis*. *S. epidermidis* is normally a human skin commensal bacterium but turns into a very common nosocomial infection pathogen to immunocompromised patients with implanted medical devices (Otto, 2009 ▶; Ziebuhr *et al.*, 2006 ▶). The therapeutic challenge of treating the *S. epidermidis* infections originates from its rapid development of antibiotic resistance and formation of biofilm (Otto, 2009 ▶). Upcoming multiresistancy to several *S. epidermidis* strains was connected to horizontal gene transfer, which is commonly mediated by bacteriophages. One of those phages is CNPH82, a member of the Siphoviridae family and the Caudovirales order.

Transmission electron microscopy micrographs showed that CNPH82 contains an isometric head and noncontractile tail (Daniel *et al.*, 2007 ▶). The complete genome of CNPH82 has been sequenced (Daniel *et al.*, 2007 ▶). However, unlike the well characterized double-stranded DNA bacteriophages such as T4, T7, P22 and SPP1, no X-ray structural information has yet been deduced for proteins of this essential pathogen-related phage.

The portal protein serves as a major component of the ATP-dependent genome translocation molecular motor in tailed bacterio­phages and herpes viruses (Casjens, 2011 ▶). As an essential requirement during the viral morphogenesis process, the portal protein plays indispensable roles in several aspects: it initiates pro-capsid assembly, and is a central component of the DNA translocation molecular motor, headful sensor and connector assembly (Rao & Feiss, 2008 ▶). The portal proteins from different tailed bacteriophages and herpes simplex viruses vary dramatically in both amino acid sequence and molecular mass, but share common characteristics: cyclical homo-oligomers arranged radially with a turbine-like shape and a central channel for DNA passage (Orlova *et al.*, 1999 ▶; Rao & Feiss, 2008 ▶). In the functional mature viron or in the isolated connector bound to tail factors, portal proteins were consistently presented as 12-subunit assemblies (Olia *et al.*, 2011 ▶; Orlova *et al.*, 2003 ▶; Simpson *et al.*, 2000 ▶). Nevertheless, the oligomeric state of portal proteins from some viruses, like SPP1 and herpes virus, could change to 13 when heterologously expressed in *Escherichia coli*, possibly due to conformational rearrangements (Orlova *et al.*, 2003 ▶; Lebedev *et al.*, 2007 ▶; Cardone *et al.*, 2007 ▶; Trus *et al.*, 2004 ▶). Each subunit of SPP1 portal protein consists of four regions termed the clip, stem, wing and crown (Lebedev *et al.*, 2007 ▶). CNPH82 portal protein shares 32% amino-acid sequence identity with the SPP1 portal protein. Moreover, high sequence similarity between other head morphogenesis proteins such as the major capsid and scaffolding proteins of CNPH82 and SPP1 implies similar morphogenesis processes.

## 2. Materials and methods

### 2.1. Cloning, expression and purification of CNPH82 portal protein

The partial gene encoding truncated portal protein cn3 (E25-Q456) was amplified by PCR and ligated into the *Nhe*I/*Hin*dIII sites of vector pET28a (Novagen). Sequencing and alignment were performed to confirm the correct insert. The portal protein cn3 with a cleavable N-terminal hexahistidine tag was overexpressed in *E. coli* strain B834 cells. Cells were grown in Luria–Bertani medium with 30 µg ml^−1^ kanamycine at 310 K to the mid-log phase (OD_600_ around 0.6–0.8). The portal protein expression was induced by the addition of 1 m*M* IPTG carried out for 20 h at 289 K. The cell pellet was lysed using a cell disruptor (Constant Cell Disruption Systems) at 277 K with a pressure of 25 kPa in lysis buffer containing 20 m*M* Tris (pH 7.5), 500 m*M* NaCl, 10 m*M* MgCl_2_, 10 m*M* imidazole, 100 µg ml^−1^ lysosome, 1 m*M* 4-(2-aminoethyl) benzenesulfonyl fluoride hydrochloride (ABESF), 0.7 µg ml^−1^ pepstatin. Nickel affinity chromatography was performed on a 5 ml HiTrap chelating HP column (GE Healthcare) and the protein sample was further purified on a Superose 6 size-exclusion column (GE Life Sciences). Purity was assigned by denaturing polyacrylamide gel electrophoresis. The molecular mass of the purified sample was confirmed by matrix-assisted laser desorption/ionization mass spectrometry (MALDI–MS).

### 2.2. Molecular weight determination by size-exclusion chromatography–multi-angle laser light scattering (SEC–MALLS)

The molecular mass of cn3 (E25-Q456) was determined by size-exclusion chromatography coupled with multi-angle laser light scattering (SEC–MALLS). The protein sample (60 µl) with a concentration of 0.5 mg ml^−1^ was applied on a BioSep SEC-s3000 gel filtration column (Phenomenex) equilibrated with buffer containing 20 m*M* Tris (pH 7.5), 500 m*M* NaCl, 10 m*M* MgCl_2_. Size-exclusion chromatography was carried out on a Shimadzu HPLC system and the elution was monitored at 280 nm by an SPD20A UV/Vis detector. Light-scattering data were recorded by a Dawn HELEOS-II 18-angle light-scattering detector and the concentration of the eluting protein was measured by an in-line Optilab rEX refractive-index monitor (Wyatt Technology). Data were analysed with the *ASTRA V* software package (Wyatt Technology). Molecular mass was calculated using Zimm’s formalism of the Rayleigh–Debye–Gans light-scattering model for dilute polymer solutions and a refractive-index increment (d*n*/d*c*) of 0.183 ml g^−1^ was used for the protein molecular mass estimation.

### 2.3. Crystallization

The protein cn3 (E25-Q456) was crystallized at 293 K by the sitting-drop vapour diffusion method using 15 mg ml^−1^ protein solution in 20 m*M* Tris (pH 7.5), 500 m*M* NaCl, 10 m*M* MgCl_2_. Drops containing 300 nl cn3 solution and 150 nl reservoir solution were dispensed by a Mosquito Nanolitre Pipetting robot (TTP Lab-tech) and equilibrated against 60 ul of reservoir solution. To overcome the hurdle of high salt concentration in the protein solution, 500 m*M* NaCl was added into the reservoir solution after the screen was set up. The best crystal was obtained with the reservoir containing 0.2 *M* ammonium acetate and 40%(*v*/*v*) 2-methyl-2,4-pentanediol (MPD).

### 2.4. X-ray data collection and processing

X-ray data were collected from a single crystal at the ESRF beamline ID14-4 at a wavelength of 0.9393 Å with a crystal-to-detector distance of 652.7 mm. Data were collected at 100 K using an oscillation range of 0.5° per image with a total crystal rotation of 180°. Diffraction images were indexed and integrated using *HKL*-2000 (Otwinowski & Minor, 1997 ▶) and were further analysed with the *CCP4* program package (Winn *et al.*, 2011 ▶). The self-rotation function was calculated using *MOLREP* (Vagin & Teplyakov, 2010 ▶), in the resolution range 5–10 Å with the radius of integration sphere of 87 Å. To solve the structure by molecular replacement, *BALBES* (Long *et al.*, 2008 ▶), *MOLREP* (Vagin & Teplyakov, 2010 ▶) and *Phaser* (McCoy *et al.*, 2007 ▶) were tried and SPP1 portal protein gp6 was used as a search model (PDB access code 2jes).

## 3. Results and discussion

### 3.1. Cloning, expression and purification

The portal protein was cloned and over-expressed in *E. coli* B834 cells. Homogeneous protein was obtained after Ni affinity and size-exclusion chromatography. The protein was concentrated to ∼35 mg ml^−1^ in solution containing 20 m*M* Tris (pH 7.5), 500 m*M* NaCl, 10 m*M* MgCl_2_.

### 3.2. Oligomeric state of CNPH82 portal protein cn3

**Figure 1:**
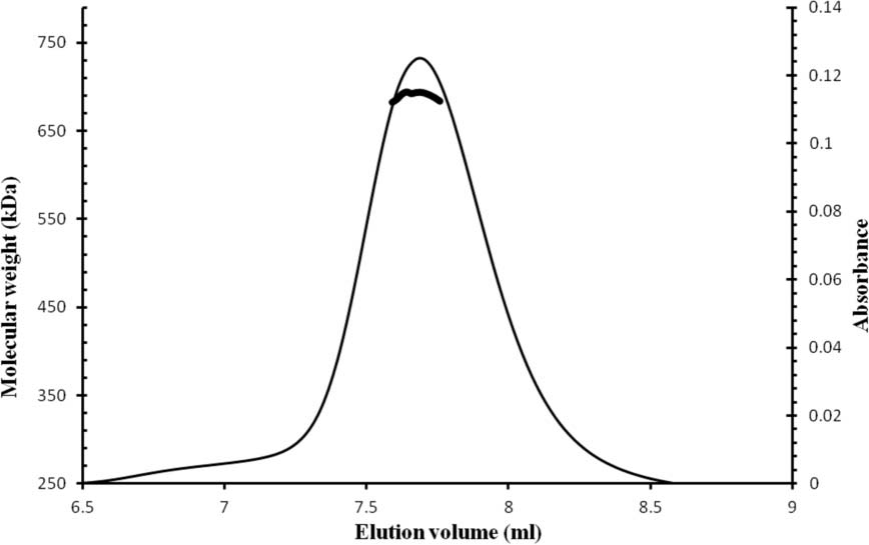
Characterization of cn3 oligomeric state by SEC–MALLS. The thin line corresponds to the absorbance monitored at 280 nm. The thick line shows the molecular weight calculated for the eluted species.

The truncated CNPH82 portal protein cn3 (E25-Q456) consists of 432 amino acids with a theoretical molecular mass of 53.074 kDa. The molecular weight of the purified protein measured by MALDI–MS is 53.094 kDa, in good agreement with the theoretical value. A single monodisperse peak was observed during the size-exclusion chromatography of cn3. SEC–MALLS showed the mean molecular weight of the eluted species to be 685.9 kDa, or ∼12.9 subunits per oligomer, suggesting that cn3 contains 13 subunits per oligomer in solution (Fig. 1 ▶).

### 3.3. Crystallization of CNPH82 portal protein cn3

**Figure 2:**
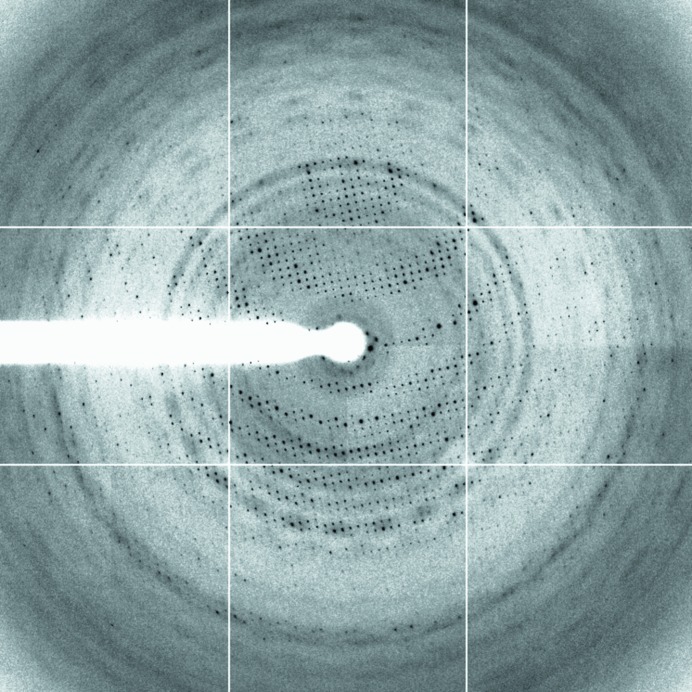
Diffraction image. Resolution at the edge of the plate is 3.9 Å.

Several hits appeared in the initial MPD crystallization screen (Hampton) with the best diffracting crystals growing from 40% MPD containing either 0.2 *M* ammonium nitrate or 0.2 *M* ammonium acetate. Both conditions were optimized. A complete native data set to a resolution of 4.2 Å was collected at the ESRF using a crystal grown from 0.2 *M* ammonium acetate and 40%(*v*/*v*) MPD (Fig. 2 ▶).

### 3.4. Crystallographic analysis reveals a 13-fold symmetry in the CNPH82 portal protein structure

**Figure 3:**
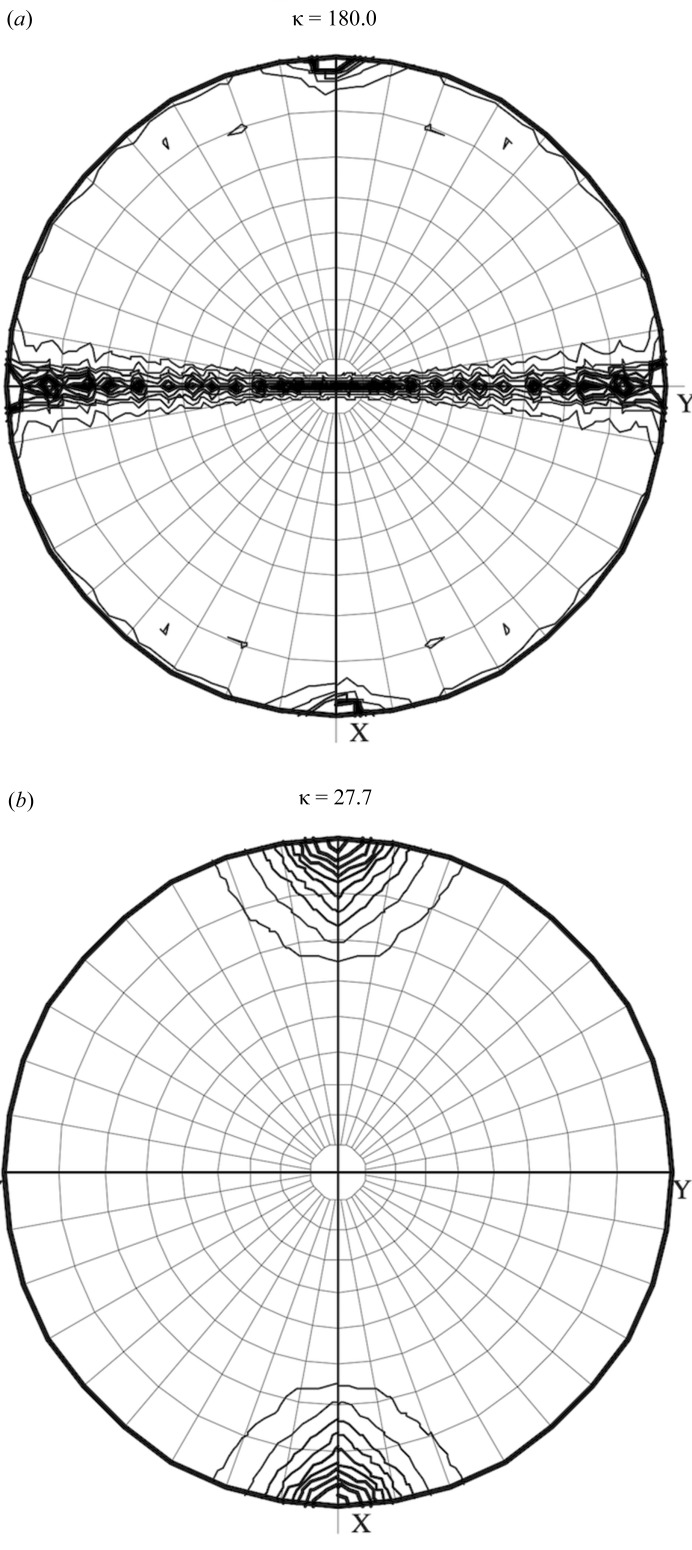
Stereographic projections κ = 180° (*a*) and κ = 27.7° (*b*) of the self-rotation function.

The crystal belongs to the space group *C*222_1_, with *a* = 252.4, *b* = 367.0, *c* = 175.5 Å (Table 1 ▶). The self-rotation function *R* (Φ, Ψ, K) (Crowther, 1972 ▶) was calculated to deduce the internal symmetry of the CNPH82 portal protein. The 13-fold symmetry was identified from peaks appearing in κ sections 360°/13 and κ = 180° (Fig. 3 ▶). Peaks in the κ = 180° section were spaced from each other by 27.7° (Fig. 3 ▶ *a*). Although the sequence identity between cn3 and the portal protein, gp6, of SPP1 is as high as 32%, attempts to solve the structure by molecular replacement proved unsuccessful.

In conclusion, the truncated portal protein cn3 of bacteriophage CNPH82 was successfully purified and crystallized. The X-ray data set collected from a native crystal was to the resolution of 4.2 Å. The oligomeric state was characterized to be 13 mer by SEC–MALLS and crystallographic analysis. Elucidating the structure of the portal protein will provide insights into the phage assembly, in particular the mechanism of viral DNA encapsidation.

## Figures and Tables

**Table 1:** X-ray data statistics Values in parentheses are for the highest-resolution shell.

X-ray source	ID14-4, ESRF
Wavelength (Å)	0.9393
Temperature (K)	100
Space group	*C*222_1_
Unit-cell parameters (Å)	*a* = 252.4, *b* = 367.0, *c* = 175.5
Resolution range (Å)	100–4.2 (4.35–4.20)
No. of unique reflections	54776 (4970)
*R*_merge_ † (%)	12.6 (65.4)
Completeness (%)	98.1 (90.1)
Redundancy	3.6 (2.9)
Average *I*/σ(*I*)	8.2 (1.4)

† *R*_merge_ = 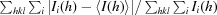, where 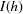 is the intensity of reflection *h*, 〈
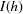
〉 is the average value of the intensity, the sum ∑*_hkl_* is over all measured reflections and the sum ∑_*i*_ is over *i* measurements of a reflection.
